# The Same Air

**DOI:** 10.3201/eid1403.079999

**Published:** 2008-03

**Authors:** Al Zolynas

—for Guy Murchie, The Seven Mysteries of Life

The same air

that moves

through me and you

through the waving branches

of the bronchial tree

through veins

through the heart

the same air

that fills balloons

that carries voices

full of lies and truths

and half-truths

that holds up the wings of butterflies

humming birds eagles hang gliders 747s

the same air

that sits like a dull relative

on humid lakes

in Minnesota in summer

the same air

trapped in vintage champagne

in old bicycle tires lost tennis balls

the air inside a vial in a sarcophagus

in a tomb in a pyramid

buried beneath the sand

the same air

inside your freezer

wrapping its cold arms

around your t.v. dinners

the same air that supports you

that supports me

the same air that moves through us

that we move through

the same air frogs croak with

cattle bellow with

monks meditate with and on

the same air we moan with

in pleasure or in pain

the breath I’m taking now

will be in China in two weeks

my lungs have passed an atom

of oxygen that passed through the lungs

of Socrates or Plato

or Lao-tsu or Buddha

or Walt Disney or Ronald Reagan

or a starving child in Somalia

or certainly you

you right here right now

yes certainly you

the same air

the very same air

